# Association of retroperitoneal fibrosis with malignancy and its outcomes

**DOI:** 10.1186/s13075-021-02627-3

**Published:** 2021-09-26

**Authors:** Sang Jin Lee, Jung Su Eun, Min Jung Kim, Yeong Wook Song, Young Mo Kang

**Affiliations:** 1 Division of Rheumatology, School of Medicine, Kyungpook National University, 130 Dongdeok-ro, Jung-gu, Daegu, 41944 Republic of Korea; 2 Department of Molecular Medicine and Biopharmaceutical Sciences, Graduate School of Convergence Science and Technology, and College of Medicine, Medical Research Institute, Seoul National University, Seoul, Republic of Korea; 3 Division of Rheumatology, College of Medicine, Seoul National University, Seoul, Republic of Korea

**Keywords:** Retroperitoneal fibrosis, Malignancy, Standardized incidence ratios, Survival

## Abstract

**Introduction:**

Retroperitoneal fibrosis (RPF) is characterized by a highly fibrotic retroperitoneal mass and encompasses the idiopathic form and secondary to malignancies. Because we have limited knowledge whether RPF is associated with malignancy, we aimed to investigate the relationship between RPF and malignancy and to compare the characteristics and prognosis of cancers among patients with RPF.

**Methods:**

Medical records of 111 patients diagnosed as having RPF were reviewed and 38 cases of cancer, confirmed by biopsy, were identified. Standardized incidence ratios (*SIR*s) were calculated for cancers and stratified according to cancer type and RPF-cancer diagnosis interval. Cancer characteristics and outcomes were compared between RPF-cancer diagnosis intervals.

**Results:**

The average age at RPF diagnosis was 59.2 ± 15.0 years, and 69.4% of the patients were male. The cancer *SIR*s in patients with RPF relative to age- and sex-matched individuals in the general population was 2.2 (1.6–3.1). *SIR*s of renal pelvis cancer and multiple myeloma were significantly higher than in the general population. When stratified by RPF-cancer intervals, the *SIR* for cancer was 9.9 within 1 year of RPF diagnosis, while no significant increase in the *SIR* was found after 1 year from RPF diagnosis. Cancer stage was more advanced at the time of diagnosis in patients within a 1-year interval for RPF than those with cancer within a >5-year interval, with a correspondingly increased mortality in the former patients.

**Conclusions:**

RPF was significantly associated with malignancy, particularly those diagnosed within 1 year of RPF diagnosis. Cancer stages at diagnosis were more advanced and the mortality rate was higher in patients within a 1-year interval between RPF and cancer diagnosis than in those with a >5-year interval between diagnoses.

**Supplementary Information:**

The online version contains supplementary material available at 10.1186/s13075-021-02627-3.

## Introduction

Retroperitoneal fibrosis (RPF) is a rare condition characterized by the presence of chronic inflammation and fibrotic retroperitoneal tissue, which often wraps around the aorta and causes ureteral obstruction [1]. The idiopathic form of the disease encompasses more than two-thirds of cases, with the remaining cases occurring secondary to malignancy, infection, radiotherapy, surgery, or drugs [2, 3]. The idiopathic form may be associated with primary large vessel inflammatory diseases because it involves the thoracic aorta and epiaortic arteries as well as the abdominal aorta in one-third of patients [4]. About half of the idiopathic form was found to be in the spectrum of immunoglobulin G4-related diseases (IgG4-RDs) showing more than 40% of IgG4/IgG ratio in histopathologic features [5, 6]. However, the identification of secondary causes such as malignancy is often difficult in clinical practice [7].

IgG4-RD, which presents as mass lesions in the pancreas, retroperitoneum, kidney, salivary/lacrimal gland, and lung, among others, is a systemic inflammatory and sclerosing condition with IgG4+ plasma cell infiltration in affected tissues [8]. Depending on the investigated IgG4-RD cohorts, IgG4-RD is accompanied by RPF involvement in 9.6% of cases excluding periaortitis [9] to 56% of cases including periaortitis [10]. IgG4-RD patients with RPF generally exhibit diffuse IgG4-positive plasma cell infiltration and an IgG4/IgG+ plasma cell ratio of >40% in their retroperitoneal tissue [5, 10, 11], but IgG4-RD and non-IgG4 RD in RPF are not easy to distinguish by clinical appearance.

IgG4-RD was reportedly significantly associated with malignancy, particularly during the first year after IgG4-RD diagnosis [12–14]. However, after complete treatment of the accompanying cancer, the rate of relapse of IgG4-RDs such as autoimmune pancreatitis is low [14]. Furthermore, there are no reported cases of IgG4-RDs invading the organ previously affected by cancer [12, 14]. These results may indicate that some IgG4-RD can be classified as paraneoplastic syndromes.

Studies evaluating the association of RPF with malignancy are rare, but one large size (*n* = 204) study reported that 31 (15.2%) patients with RPF had malignancies [7]. However, long-term follow-up data were not collected, and a survival analysis was not performed in this study. Idiopathic RPF is often difficult to differentiate from atypical malignancy in patients, and biopsy is necessary in these patients [15, 16]. Because patients who have malignancy concurrently with secondary RPF present with a poorer prognosis, it is important to distinguish idiopathic RPF from retroperitoneal malignancy in some cases [3, 7].

We have, however, limited knowledge of the risk and characteristics of cancers that occur in patients with RPF. We thus aimed to investigate the relationship between RPF and malignancy and to compare the characteristics and prognosis of cancers among patients with RPF.

## Methods

### Enrolled patients and data collection

This study was approved by the Institutional Review Boards (IRB) of Kyungpook National University Hospital (KNUH) and Seoul National University Hospital (SNUH) in Korea (2017-02-007, H-1808-128-967, respectively). The requirement for informed consent was waived by the IRB since the study involved a minimum risk to the enrolled patients and no identifiable information was used. All methods were performed in accordance with the relevant guidelines and regulations. A total of 111 RPF patients who received medical care at KNUH and SNUH from January 1999 to December 2016 were enrolled in this retrospective study. RPF was diagnosed according to the clinical codes. Exclusion criteria included patients who were younger than 18 years old and who did not fulfill classification by evaluation of computed tomography (CT) or positron emission tomography (PET)/CT. Thirty-eight cases of cancer, confirmed by biopsy, were identified in 34 patients with RPF by evaluating all patients. Data related to demographic characteristics and malignancy ascertainment were obtained from the medical records (supplementary figure 1).

### Time interval between RPF and cancer diagnosis

Assuming that RPF developing concurrently with cancer is associated with the cancer, we intended to estimate and compare the risk of cancers and survival duration by adopting RPF-cancer diagnosis intervals. The interval was calculated using the date of RPF diagnosis as a reference. Given that a 1-year interval is used for strict definition in cancer-associated autoimmune diseases [17], we divided the patients into groups according to the intervals between RPF and cancer diagnoses. We compared a 1-year interval group, where cancer was diagnosed within 1 year of RPF diagnosis, with other groups.

### Cancer stages

Where applicable, cancer stages were based on the tumor-node-metastasis staging system suggested by the American Joint Committee on Cancer and the Union for International Cancer Control [18]. Stages of certain cancers (e.g., lymphoma, myelodysplastic syndrome, multiple myeloma, liposarcoma, and gastrointestinal stromal cancer) were assessed using staging systems specific to the respective cancer types [19, 20]. Seven of the 38 cases of cancer were not included in only analyses pertaining to cancer stages due to lack of information regarding cancer stages in the medical records.

### Patient survival

The follow-up period for each patient was defined as the period from cancer diagnosis to the latest clinic visit (or to the date of death). For those patients lost to follow-up, the Korean national mortality database (http://kostat.go.kr/portal/korea/index.action) was used to survey the date of death or to determine whether the patient was still alive as of December 31, 2016, which was considered to be the final follow-up date. Survival curves were created using the Kaplan-Meier method.

### Statistical analysis

Continuous variables are expressed as the mean ± SD and categorical variables as numbers and percentages. Comparisons were performed using either the Student’s *t*-test or Fisher’s exact test. The cancer risk was determined by standardized incidence ratios (*SIR*s) and the exact Poisson method was used to calculate the 95% confidence interval (*CI*). The *SIR* was estimated by dividing the observed number of cancer occurrences by the expected number of occurrences in our RPF patient cohort. The expected number of cancer cases for the corresponding person-year of 111 RPF patients was estimated using the calendar-year-specific cancer incidence of the age- and sex-matched Korean population (Korean National Cancer Registry data, http://kosis.kr). *SIR*s were calculated for all cancers and stratified according to the cancer type, age at cancer diagnosis, and time interval between RPF diagnosis and cancer diagnosis. Because the Korean National Cancer Registry data for cancer incidence were available for 1999–2016, the 1999 cancer incidence was used to extrapolate the expected incidence of skin cancer in 1996. To calculate *SIR*, the study period was applied from 20 years prior to the first diagnosis of RPF to the date of the last follow-up because the earliest cancer diagnosis time among all cancer cases in this study was −20 years from the date of the first diagnosis of RPF. Age- and sex-adjusted *HR*s for mortality were calculated using the Cox proportional hazards model for the survival curves. *p* values of ≤ 0.05 were considered statistically significant. All statistical analyses were performed using IBM SPSS version 19, and graphics were generated with GraphPad Prism.

## Results

### Baseline characteristics of patients with RPF

The flow chart for this study is shown in supplementary figure 1. Among the 111 patients with RPF, the mean age at RPF diagnosis was 59.2 ± 15.0 years and the mean follow-up duration after RPF diagnosis was 9.3 ± 3.6 years. The sites of RPF in the retroperitoneal space included the aorta, which manifested as periaortitis (94 cases, 84.7%), and the retroperitoneal soft tissue, which manifested as a tumor-like mass (17 cases, 15.3%). Among the 94 cases of periaortitis, four exhibited multifocal periaortitis, including two cases at the descending aorta and one case each at the ascending aorta and celiac axis. RPF was also observed in systemic organs including the lymph nodes (18 cases, 16.2%), kidneys (13 cases, 11.7%), pancreas (9 cases, 8.1%), salivary glands (5 cases, 4.5%), and lacrimal glands (1 case, 0.9%). Sixty-six cases (59.5%) presented with hydronephrosis, and the mean serum creatinine levels were 2.0 ± 3.1 mg/dL. The mean IgG (*n* = 46) and IgG4 (*n* = 51) concentrations were 1522.3 ± 564.2 and 302.7 ± 625.4 mg/dL, respectively (Table 1).

**Table 1:** Baseline characteristics of 111 patients with RPF

Baseline characteristics	*n* = 111	
Age at diagnosis of RPF (years)	59.2 ± 15.0	
Mean follow-up duration (years) after RPF diagnosis	9.3 ± 3.6	
Male, *n* (%)	77 (69.4)	
Retroperitoneal distributions, *n* (%)		
Periaortitis^a^	94 (84.7)	
Retroperitoneal tumor-like mass	17 (15.3)	
Systemic organ involvement, *n* (%)		
Lymph nodes	18 (16.2)	
Kidney	13 (11.7)	
Pancreas	9 (8.1)	
Salivary gland	5 (4.5)	
Lacrimal gland	1 (0.9)	
Hydronephrosis, *n* (%)	66 (59.5)	
Creatinine (mg/dL) (*n* = 100)	2.0 ± 3.1	
ESR (mm/h) (*n* = 81)	47.6 ± 31.6	
CRP (mg/dL) (*n* = 90)	2.8 ± 4.4	
IgG (mg/dL) (*n* = 46)	1522.3 ± 564.2	
IgG4 (mg/dL) (*n* = 51)	302.7 ± 625.4	
IgG4 > 135 (mg/dL), *n* (%)	23/51 (45.1)	

Data are expressed as means ± SD for continuous variables or numbers and percentages for categorical variables. *RPF* retroperitoneal fibrosis; *CT* computed tomography; *PET* positron emission tomography; *ESR* erythrocyte sedimentation rate; *CRP* C-reactive protein; *IgG* immunoglobulin G. ^a^Four cases (two cases of descending aorta and one case each of ascending aorta, celiac axis aortitis) were found at the extra-aortic bifurcation

Thirty-eight cases of cancer were identified in 34 patients with RPF. RPF patients with malignancies were significantly older than those without malignancies at the time of RPF diagnosis (64.2 ± 12.1 versus 57.0 ± 15.0; *p* = 0.018) (Supplementary Table 1).

### Cancer risk in patients with RPF

**Fig. 1:**
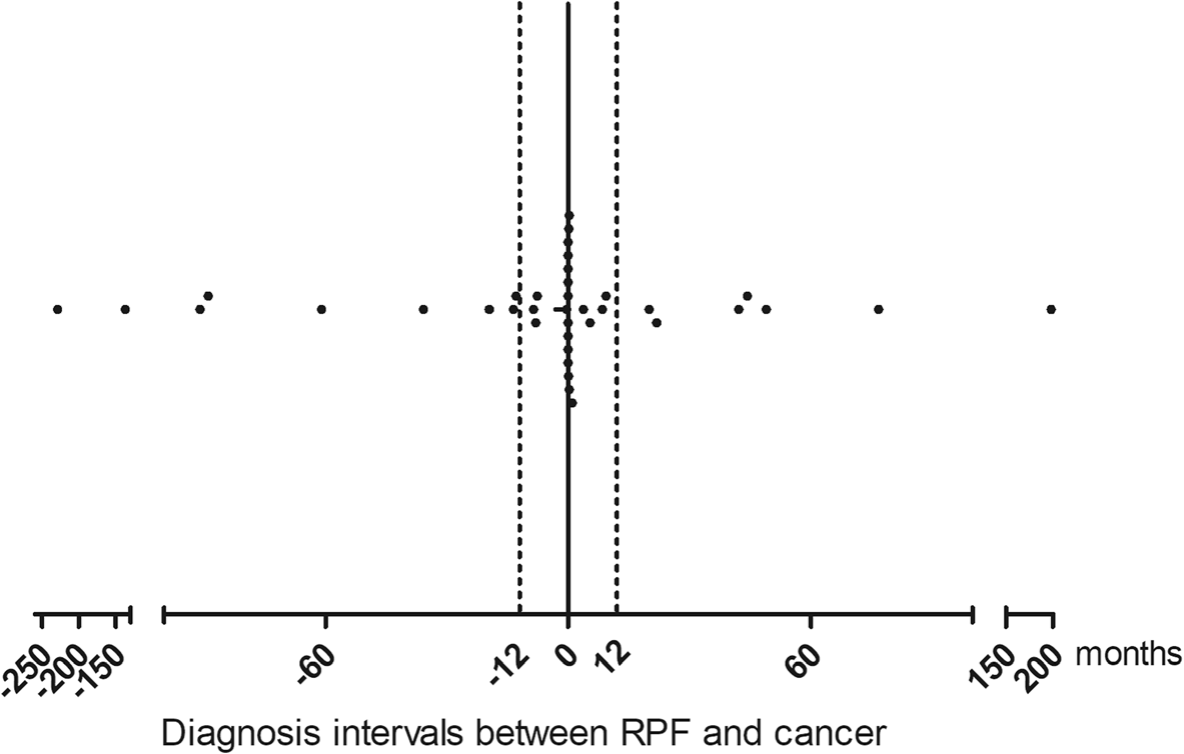
Time from RPF diagnosis to cancer diagnosis. Dot represents each case. RPF, retroperitoneal fibrosis

The cancer *SIR* in patients with RPF relative to age- and sex-matched individuals in the general population was 2.2 (*95% CI* 1.6–3.1; 1.9 [*95% CI* 1.2–2.8] in men; 3.5 [*95% CI* 1.8–6.2] in women). Cancer *SIR*s were the highest in those aged 40–49 years (4.4 [*95% CI* 1.2–11.2]), followed by those aged 50–59 years (2.8 [*95% CI* 1.2–5.6]). The mean interval between RPF and cancer diagnosis was 31.4 ± 54.1 months. When stratified by intervals between RPF and cancer diagnoses, cancer *SIR* within 1 year of RPF diagnosis was significantly higher than that of the general population (9.9 [*95% CI* 6.2–15.0]); however, there was no significant elevation of *SIR*s in other interval groups beyond 1 year after RPF diagnosis (Table 2). In order to display the distribution of intervals between RPF and cancer diagnoses, we expressed the intervals using a dot graph, which shows the pyramidal distribution with the peak at the time of RPF diagnosis (Fig. 1). The highest *SIR*s were for renal pelvis cancer (74.4 [*95% CI* 15.4–217.5]), followed by multiple myeloma (19.0 [*95% CI* 2.3–68.5]) (Table 2).

**Table 2:** Standardized incidence ratios (*SIR*s) of cancers in patients with RPF

	Observed	Expected	*SIR* [*95% CI*]	*Corrected p*
Overall *SIR*	38	17.1	2.2 [1.6, 3.1]	<0.001
Male	26	13.7	1.9 [1.2, 2.8]	<0.001
Female	12	3.4	3.5 [1.8, 6.2]	<0.001
Age-specific *SIR*				
30–39	1	0.23	4.3 [0.1, 23.7]	0.419
40–49	4	0.91	4.4 [1.2, 11.2]	0.028
50–59	8	2.83	2.8 [1.2, 5.6]	0.017
60–69	11	6.58	1.7 [0.8, 3.0]	0.143
70–79	12	5.48	2.2 [1.1, 3.8]	0.022
≥ 80	2	1.00	2.0 [0.2, 7.2]	0.529
Interval-specific *SIR*, years^a^				
−20 ~ −5	5	6.47	0.77 [0.3, 1.8]	0.747
−5 ~ −3	0	1.95	0	–
−3 ~ −1	4	2.26	1.8 [0.5, 4.6]	0.365
−1 ~ +1	22	2.22	9.9 [6.2, 15.0]	<0.001
+1 ~ +3	2	1.33	1.5 [0.2, 5.4]	0.771
+3 ~ +5	3	0.83	3.6 [0.8, 10.6]	0.102
+5 ~	2	2.02	1.0 [0.1, 3.6]	1.000
Cancer type-specific *SIR*				
Lung	4	2.7	1.5 [0.4, 3.9]	0.555
Stomach	6	3.3	1.8 [0.7, 4.0]	0.228
GIST	1	0.06	16.4 [0.4, 91.2]	0.119
Colon	3	1.26	2.4 [0.5, 7.0]	0.268
Rectum	1	1.05	1.0 [0.0, 5.3]	1.000
Pancreas	2	0.49	4.1 [1.0, 14.8]	0.174
Kidney	2	0.31	6.4 [0.8, 23.2]	0.079
Renal pelvis	3	0.04	74.4 [15.4, 217.5]	<0.001
GB and biliary tract	1	0.53	1.9 [0.1, 10.6]	0.818
Bladder	1	0.46	2.2 [0.1, 12.1]	0.739
Prostate	1	1.04	1.0 [0.0, 5.4]	1.000
MM	2	0.11	19.0 [2.3, 68.5]	0.010
NHL	1	0.29	3.5 [0.1, 19.4]	0.500
Connective and soft tissue	1	0.06	15.5 [0.4, 86.2]	0.125
MDS	1	0.06	16.8 [0.4, 93.5]	0.116
Thyroid	2	0.74	2.7 [0.3, 9.8]	0.339
Breast	1	0.41	2.4 [0.1, 13.4]	0.679
Cervix uteri	1	0.18	5.5 [0.1, 30.9]	0.330
Larynx	1	0.19	5.3 [0.1, 29.7]	0.342
Skin^b^	1	0.25	3.9 [0.1, 21.9]	0.449
Unknown primary	2	0.20	9.8 [1.2, 35.4]	0.037

Data are expressed as *SIR* (*95% CI*). *RPF* retroperitoneal fibrosis; *GIST* gastrointestinal stromal tumor; *GB* gall bladder; *MM* multiple myeloma; *NHL* non-Hodgkin lymphoma; *MDS* myelodysplastic syndrome. ^a^The interval was calculated using the data of RPF diagnosis as a reference. ^b^Given that the Korean National Cancer Registry data were available between 1999 and 2016, the 1999 cancer incidence was used to extrapolate the expected cancer occurrence for a skin cancer that was diagnosed in 1996

### Characteristics of cancers in patients with RPF

When cancers were analyzed according to organ involvement, the stomach (*n* = 6) was the most common site, followed by the lung (*n* = 4), colon (*n* = 3), and renal pelvis (*n* = 3). The number of patients who were diagnosed with cancer within 1 year of RPF diagnosis was 22/38 (57.9%). Cancers involving retroperitoneal organs including the renal pelvis, pancreas, and kidney parenchyma were exclusively diagnosed in the 1-year interval group. The predominant cellular origin was the epithelial cells, which included adenocarcinoma (*n* = 18) and transitional cell carcinoma (*n* = 4). Cancers that simultaneously developed with RPF onset had a more advanced staging, and RPF newly developed in two patients while the cancer worsened and spread (case numbers 14 and 18) (Table 3).

**Table 3:** Characteristics of the retroperitoneal fibrosis patients with malignancies (*n* = 38)

Case	Sex	Age at RPF diagnosis	Age at malignancy diagnosis	Malignancy site	Malignancy type	Stage (TNM)
1	M	68	68	Lung	Adenocarcinoma	I
2	M	76	76	Lung	NSCLC	IV
3	M	52	52	Stomach	Adenocarcinoma	IV
4	M	67	67	Stomach	Adenocarcinoma	IV
5	F	82	82	Stomach	Adenocarcinoma	–
6	M	68	67	Small intestine	GIST	IV
7	M	49	49	Colon	Adenocarcinoma	IIA
8	M	52	51	Colon	Adenocarcinoma	IIIB,
9	M	54	54	Pancreas	Adenocarcinoma	–
10	M	78	78	Pancreas	Adenocarcinoma	–
11	M	47	46	Kidney	Clear cell	II
12	M	64	64	Kidney	Clear cell	I
13	M	51	51	Renal pelvis	TCC	IV
14*	F	79	79	Renal pelvis	TCC	III
15	F	77	78	Renal pelvis	TCC	IV
16	F	75	75	GB	Adenocarcinoma	–
17	M	67	67	Bladder	TCC	I
18†	F	67	67	Bone	Adenocarcinoma	IV
19	M	74	74	Prostate	Adenocarcinoma	II
20	M	73	73	Peritoneum	MM	III
21	F	64	64	Peritoneum	NHL	III
22	F	75	75	Lymph node	Squamous	IV
23	F	50	49	Thyroid	PTC	I
24‡	M	67	69	Lung	Adenocarcinoma	I
25	M	53	51	Stomach	Signet ring	I
26	M	68	67	Colon	Adenocarcinoma	IIIB
27§	M	78	80	Rectum	Adenocarcinoma	–
28	M	40	43	Peritoneum	Liposarcoma	IIIB
29	F	42	39	Cervix uteri	Adeno	II
30	M	49	52	BM	MDS	–
31‡	M	67	71	BM	MM	–
32†	F	67	62	Breast	Adenocarcinoma	II
33	F	66	72	Thyroid	PTC	II
34¶	M	71	63	Larynx	Squamous	IV
35¶	M	71	60	Lung	Squamous	I
36	M	65	82	Stomach	Adenocarcinoma	I
37§	M	78	71	Stomach	Adenocarcinoma	I
38	F	64	45	Skin	Basal cell	I

*RPF* retroperitoneal fibrosis; *NSCLC* non-small cell lung cancer; *GIST* gastrointestinal stromal tumor; *TCC* transitional cell carcinoma; *GB* gall bladder; *BM* bone marrow; *NHL* non-Hodgkin lymphoma; *PTC* papillary thyroid cancer; *MDS* myelodysplastic syndrome; *MM* multiple myeloma. ^*^,†RPF newly developed while the cancer worsened and spread. †,‡,§,¶Those cases were the same patient. RPF and cancer were diagnosed within a 1-year interval in 22 cases (case numbers 1–22) and a greater than 5-year interval in 7 cases (case numbers 32–38)

### Staging at cancer diagnosis when stratified by RPF-cancer diagnosis interval

**Fig. 2:**
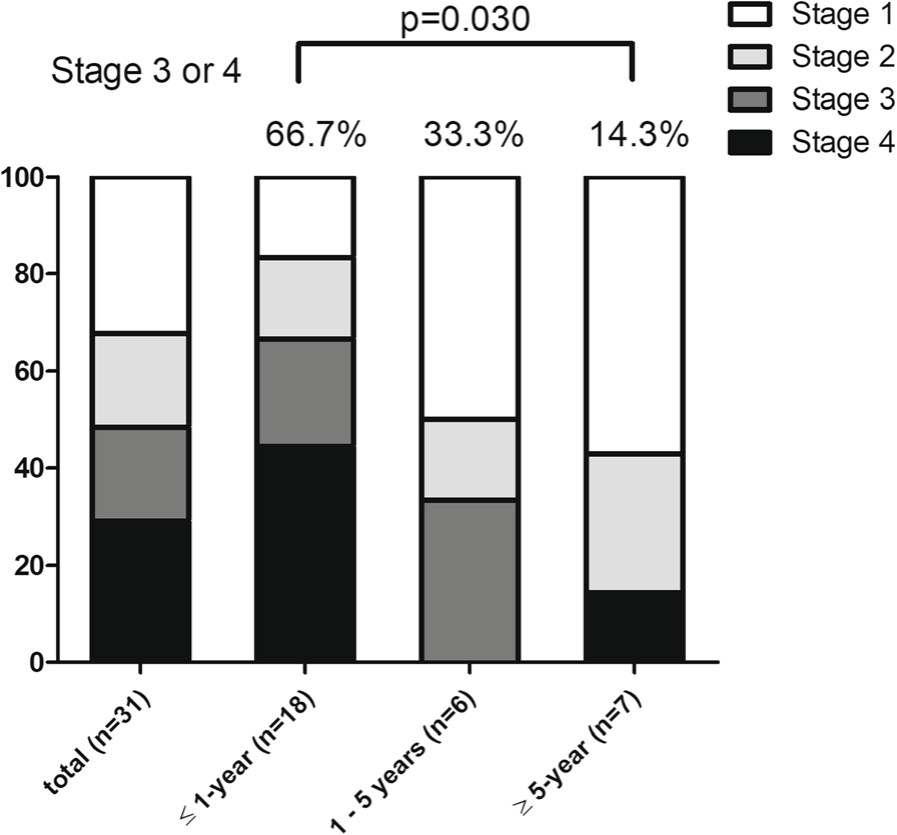
Cancer stage at cancer diagnosis when stratified by RPF-cancer diagnosis intervals. When stratified by RPF-cancer diagnosis intervals, cancers revealed more advanced stages in patients whose RPF and cancer were diagnosed within a 1-year interval compared to those with a greater than 5-year interval. The numbers in brackets denote cancer cases. The comparison was performed using Fisher’s exact test. RPF, retroperitoneal fibrosis

Seven patients with cancer were excluded from the staging analysis due to lack of information regarding staging in the medical records. Of the 31 malignancies with staging information, the frequencies of stages I, II, III, and IV were 10 (32.2%), six (19.4%), six (19.4%), and nine (29.0%), respectively. The proportion of patients with advanced cancer stages (stages III and IV) was significantly higher among patients who received diagnoses of RPF and cancer within a 1-year interval than among those with a >5-year interval (66.7 versus 14.3%, *p* = 0.030) (Fig. 2). The stages of cancers in specific organs were demonstrated according to the RPF-cancer diagnosis intervals, which revealed that the proportion of patients with advanced stages was higher in the 1-year interval group than in the other groups (Supplementary Figure 2).

### Prognosis of patients with cancers when stratified by RPF-cancer diagnosis interval

**Fig. 3:**
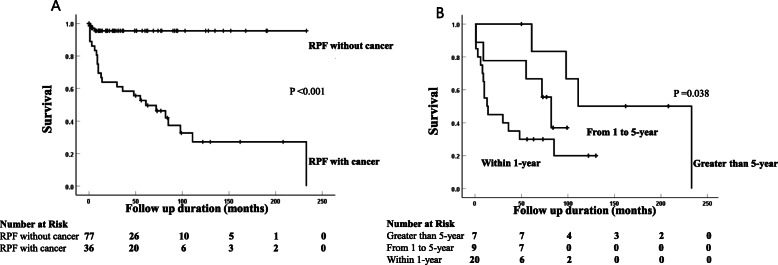
Survival analysis in patients with and without cancers when stratified by RPF-cancer diagnosis intervals. Survival time in all patients with and without cancer (**A**). When stratified by RPF-cancer diagnosis intervals, survival time in patients whose RPF and cancer were diagnosed within a 1-year interval was significantly shorter than in those with a greater than 5-year interval by Kaplan-Meier analysis (*p* = 0.038) (**B**). Survival analysis was applied for 36 cases of cancers

Given that the autoimmune disease that manifests as paraneoplastic syndrome is associated with a higher burden of tumors that resulted in poor prognosis [13, 14, 17], we analyzed the survival curve according to the RPF-cancer diagnosis interval. While 34 RPF patients with cancer died as a result of cancer progression (*n* = 18), infection (*n* = 2), or acute myocardial infarction (*n* = 1), 77 RPF patients without cancer died as a result of infection (*n* = 2) or acute myocardial infarction (*n* = 1) (Fig. 3A). Using Kaplan-Meier analysis, the mean survival time was 44.2 (*95% CI* 22.1–66.3) months in patients within a 1-year interval and 180.5 (*95% CI* 124.2–236.8) months in patients with a >5-year interval. The age- and sex-adjusted *HR* of mortality was significantly higher in patients within a 1-year interval than in those with a >5-year interval (3.9 [*95% CI* 1.1–13.9], *p* = 0.038) (Fig. 3B).

## Discussion

Although a subset of RPF cases manifest as paraneoplastic syndrome, information regarding the relationship between RPF and cancer is unclear. The present study analyzed the incidence, pathologic type, stage, and prognosis of tumors in RPF patients with cancer. Our study yielded two main findings: First, RPF was significantly associated with the occurrence of cancer, particularly within 1 year of RPF diagnosis. Second, the stages of cancer were more advanced at the time of cancer diagnosis in patients within a 1-year interval between RPF and cancer diagnosis than in those with a >5-year interval, which resulted in higher mortality in the former group. Our results indicate that a proportion of RPF cases were indeed cases of paraneoplastic syndromes; these cases predominantly occurred in patients with advanced malignancy.

Autoimmune diseases manifesting as paraneoplastic syndromes reportedly develop after cancer progression and improve with treatment of the concurrent cancer [14, 17]. In cases of cicatricial pemphigus, inflammatory myositis, and autoimmune pancreatitis, the relative risk for cancer was found to be significantly higher in the first year of disease, and prognosis was poor in patients with cancer who were simultaneously diagnosed with these diseases [14, 17, 21], which was consistent with our data. This may be explained by the highly mutational burden of tumors in advanced stages, which could be related to the generation of neoantigens that increase cross-reactivity with autoantigens such as those on the epidermal cell surface, regenerating muscle, and pancreas [17, 22, 23]. According to a large-scale genetic analysis, idiopathic RPF is associated with HLA-DRB1*03 and neoantigens generated by cancer may cross-reactive with autoantigen in RPF patients having HLA-DRB1*03 [24].

The most striking cancer risk was found for renal pelvis cancers (*SIR* 74.4) at advanced stages (III or IV) that occurred in the retroperitoneal region and were diagnosed within 1 year of RPF diagnosis. Because cancers involving the retroperitoneal organs such as the renal pelvis and renal parenchyma and pancreas occurred exclusively in the 1-year interval group in our study, the local effect of these cancers may play a crucial role in RPF development. Paraneoplastic autoimmunity such as cicatricial pemphigus and dermatomyositis has been associated with specific tumor pathology, such as adenocarcinoma [25, 26], which was the most frequent histological subtype in our study. It was reported that the specific IgG for phosphodiesterase 10A is a biomarker of patients with paraneoplastic neurologic autoimmune syndrome, and of these patients, 5/6 cancers were adenocarcinomas [27]. The immunologic trigger for the expression of neoantigens by the tumors has not been fully elucidated, and further studies on pathological linking are needed to investigate the association between autoantigen production and cancer development in RPF.

As indicated in our results, while the majority of patients died as a result of cancer progression (18/21) in the 34 RPF patients with cancer group, only three patients died as a result of infection (*n* = 2) or acute myocardial infarction (*n* = 1) in the 77 RPF patients without cancer group. Furthermore, cancers diagnosed within 1 year of RPF diagnosis typically presented at higher stages and were associated with higher mortality than those diagnosed at >5 years after RPF diagnosis. These findings are in line with those of previous studies reporting that RPF secondary to malignant disease is associated with a poor prognosis [28–30]. Advanced stages with tumor burden or tumor transformation are an important factor in triggering paraneoplastic autoimmune diseases such as myositis [17]. Along these lines, cancers with high burden might develop increased immunogenicity based on the exposure of tumor neoantigens to the immune system. These findings suggest that early detection of cancer in RPF patients is important and thorough examinations are required within 1 year of RPF diagnosis.

Therefore, cancer screening in Korean patients with RPF should be performed regularly at least 1 year after the diagnosis of RPF. A thorough screening for cancer development using advanced modalities such as PET/CT may be warranted based on our observational study. Recently, it has been reported that PET/CT could be applied to reduce the difficulty of differential diagnosis in retroperitoneal metastasis or malignancy mimicking RPF [15, 16]. However, cancers detected in the temporal vicinity of RPF diagnosis are typically advanced, with a vastly reduced opportunity for effective treatment. Nevertheless, a proportion of patients in whom cancer-specific biomarkers are detected may benefit from early diagnosis and treatment.

## Conclusion

In conclusion, RPF was significantly associated with the development of cancer, particularly those diagnosed within 1 year of RPF diagnosis. Cancer staging was more advanced at diagnosis and mortality was higher in patients diagnosed with cancer within a 1-year interval than in those diagnosed with cancer within a >5-year interval from RPF diagnosis.

## Supplementary Information

**Additional file 1: Supplementary Figure 1.** Flow chart of inclusion. **Supplementary Figure 2.** Individual cancer type and stages when stratified by RPF-cancer diagnosis intervals. **Supplementary Table 1.** Comparison of baseline characteristics between RPF patients with malignancies and those without.

## Abbreviations

RPF: Retroperitoneal fibrosis
IgG4-RDs: Immunoglobulin G4-related diseases
CT: Computed tomography
PET: Positron emission tomography
*SIR*s: Standardized incidence ratios
*CI*: Confidence interval
