# Supplementary material for: Association of retroperitoneal fibrosis with malignancy and its outcomes
Source: Arthritis Res Ther. 2021 Sep 26;23:249. doi: 10.1186/s13075-021-02627-3 (PMC8474894; doi:10.1186/s13075-021-02627-3)
Supplement: Supplementary file 1 — Additional file 1: Supplementary Figure 1. Flow chart of inclusion. Supplementary Figure 2. Individual cancer type and stages when stratified by RPF-cancer diagnosis intervals. Supplementary Table 1. Comparison of baseline characteristics between RPF patients with malignancies and those without. [file 13075_2021_2627_MOESM1_ESM.docx]

**Supplementary Figure 1. Flow chart of inclusion**


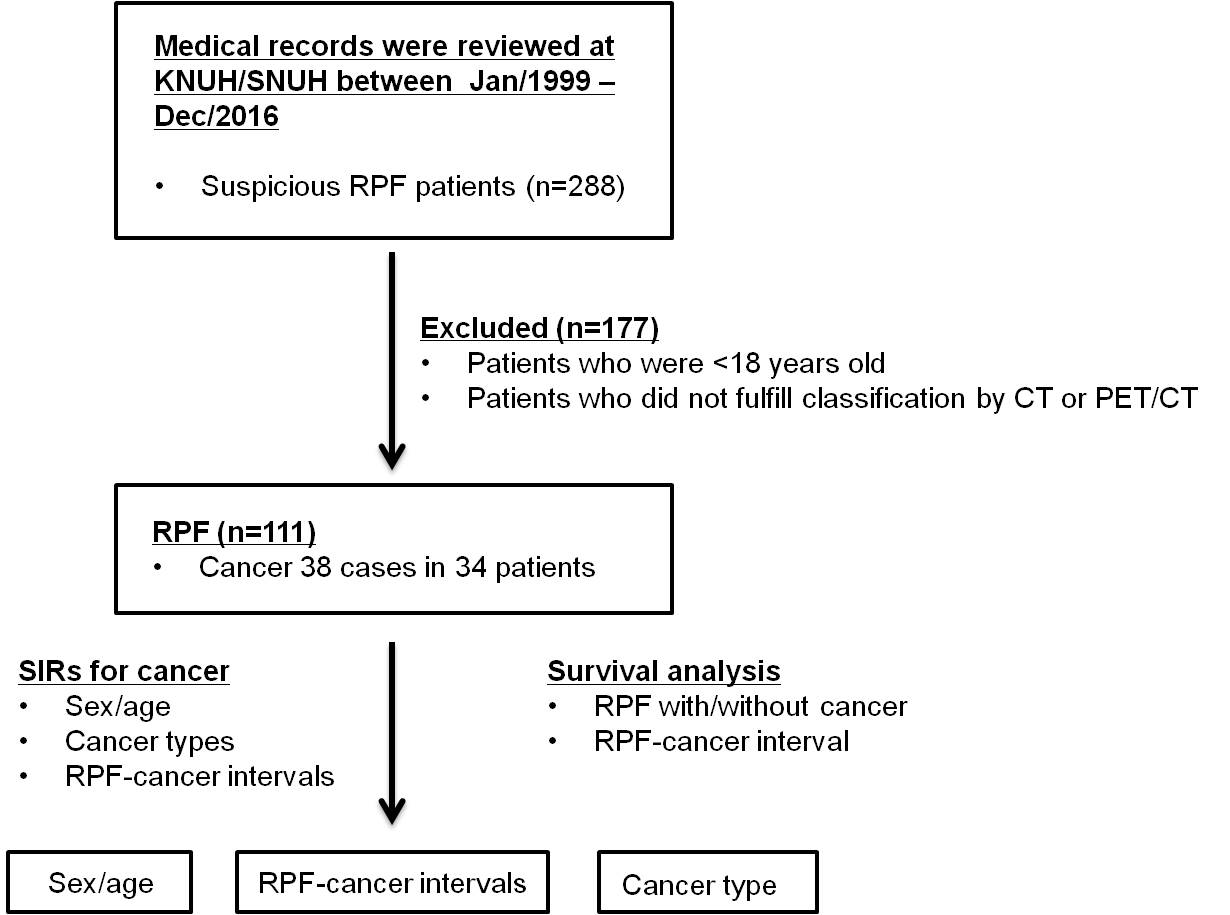


RPF, retroperitoneal fibrosis; CT, computed tomography; PET, positron emission tomography; SIRs, standardized incidence ratios.

**Supplementary Figure 2. Individual cancer type and stages when stratified by RPF-cancer diagnosis intervals**


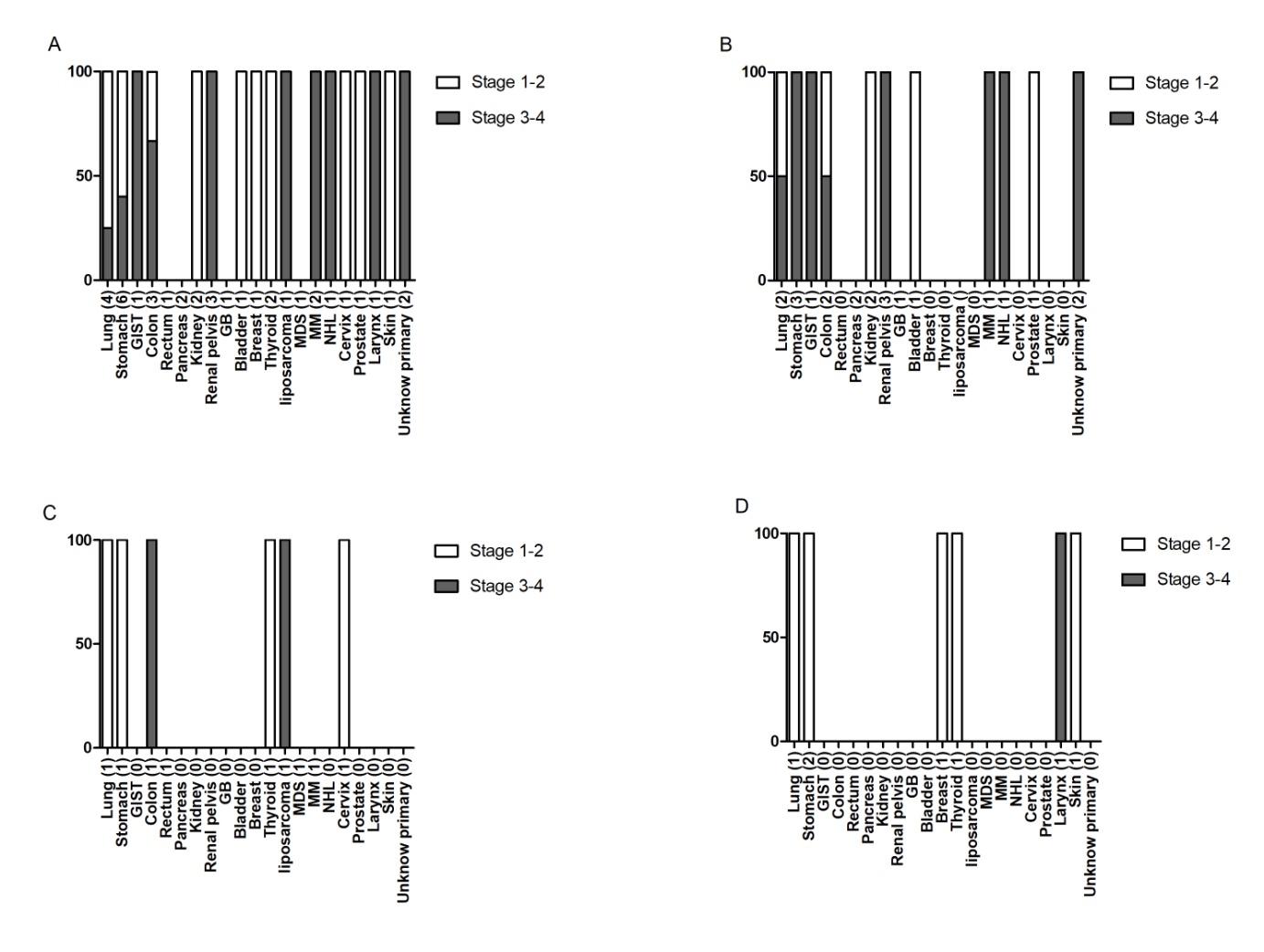


Individual cancer type and stages were shown stratified by RPF-cancer diagnosis intervals. Total cancer patients (A), cancer patients within a 1-year interval (B), from 1- to 5-year interval (C) and with greater than 5-year interval (D) between RPF and cancer diagnosis. GIST, gastrointestinal stromal tumor; GB, gall bladder; MDS, myelodysplastic syndrome; MM, multiple myeloma; NHL, non-Hodgkin lymphoma.

**Supplementary table 1. Comparison of baseline characteristics between RPF patients with malignancies and those without.**

| Baseline characteristics | Malignancy (+, n=34) | Malignancy (-, n=77) | p value |
| --- | --- | --- | --- |
| Age at diagnosis of RPF (years) | 64.2 ± 12.1 | 57.02 ± 15.0 | 0.018 |
| Male, n (%) | 23 (67.6) | 54 (70.1) | 0.794 |
| Organ involvement, n (%) |  |  |  |
| Periaortitis | 30 (88.2) | 64 (83.1) | 0.490 |
| Lymph nodes | 5 (14.7) | 13 (16.9) | 0.774 |
| Kidney | 1 (2.9) | 12 (15.6) | 0.056 |
| Pancreas† | 1 (2.9) | 8 (10.4) | 0.185 |
| Salivary gland | 2 (5.9) | 3 (3.9) | 0.642 |
| Lacrimal gland | 0 (0.0) | 1 (1.3) | 0.504 |
| Hydronephrosis, n (%) | 17 (50.0) | 49 (63.6) | 0.177 |
| Creatinine (mg/dl) (n=32, 68) | 1.7 ± 2.2 | 2.1 ± 3.4 | 0.532 |
| ESR (mm/h) (n=20, 61) | 53.6 ± 33.0 | 45.7 ± 31.2 | 0.334 |
| CRP (mg/dl) (n=25, 65) | 2.2 ± 2.7 | 3.0 ± 4.9 | 0.416 |
| IgG (mg/dl) (n=14, 32) | 1631.6 ± 593.9 | 1474.5 ± 553.6 | 0.391 |
| IgG4 (mg/dl) (n=15, 36) | 278.9 ± 400.6 | 312.7 ± 703.2 | 0.863 |
| IgG4>135 (mg/dl), n (%) | 6/15(40.0) | 17/36 (47.2) | 0.637 |

Data are expressed as means ± SD for continuous variables or numbers and percentages for categorical variables. RPF, retroperitoneal fibrosis; CT, computed tomography; PET, positron emission tomography; ESR, erythrocyte sedimentation rate; CRP, C-reactive protein; IgG, immunoglobulin G.
